# Towards an era of precise diagnosis and treatment: Role of novel molecular modification-based imaging and therapy for dedifferentiated thyroid cancer

**DOI:** 10.3389/fendo.2022.980582

**Published:** 2022-09-08

**Authors:** Jing Li, Yingjie Zhang, Fenghao Sun, Ligang Xing, Xiaorong Sun

**Affiliations:** ^1^Department of Graduate, Shandong First Medical University and Shandong Academy of Medical Sciences, Jinan, China; ^2^Department of Nuclear Medicine, Shandong Cancer Hospital and Institute, Shandong First Medical University and Shandong Academy of Medical Sciences, Jinan, China; ^3^Department of Radiation Oncology, Shandong Cancer Hospital and Institute, Shandong First Medical University and Shandong Academy of Medical Sciences, Jinan, China

**Keywords:** dedifferentiated thyroid cancer, radioactive iodine resistance, biomolecular modifications, molecular imaging, targeted therapy

## Abstract

Dedifferentiated thyroid cancer is the major cause of mortality in thyroid cancer and is difficult to treat. Hence, the essential molecular mechanisms involved in dedifferentiation should be thoroughly investigated. Several studies have explored the biomolecular modifications of dedifferentiated thyroid cancer such as DNA methylation, protein phosphorylation, acetylation, ubiquitination, and glycosylation and the new targets for radiological imaging and therapy in recent years. Novel radionuclide tracers and drugs have shown attractive potential in the early diagnosis and treatment of dedifferentiated thyroid cancer. We summarized the updated molecular mechanisms of dedifferentiation combined with early detection by molecular modification-based imaging to provide more accurate diagnosis and novel therapeutics in the management of dedifferentiated thyroid cancer.

## Introduction

Thyroid cancer (TC) is the most frequent type of cancer in the endocrine system, the incidence of which has been increasing globally in recent years (1). Although differentiated thyroid cancer (DTC) has a good prognosis, the dedifferentiated thyroid cancer, including DTC with gradual dedifferentiation, poorly differentiated thyroid cancer (PDTC) and anaplastic thyroid cancer (ATC), is the key to treatment dilemma, and leads to the death of patients.

Approximately 6-12% of DTC patients gradually lose iodine uptake ability due to dedifferentiation and eventually develop resistance to radioactive iodine (RAI) therapy, identified as RAI-refractory DTC (RAIR-DTC), demanding additional effective treatments (2, 3) The 10-year survival rate of RAIR-DTC patients with distant metastasis is only about 10% (4). PDTC and ATC account for nearly 6% and 2% of all thyroid malignancies, respectively, and usually have a poor prognosis and high mortality (5). Thus, the treatment of patients with dedifferentiated thyroid cancer remains a major clinical challenge.

In the past decade, several studies emerged and illuminated molecular mechanisms responsible for dedifferentiated thyroid cancer. The discovery of molecular modification targets has raised high hope for new potential avenues for the management of dedifferentiated thyroid cancer. In this review, there will be a focus on investigating the comprehensive and updated molecular modification-based management strategies in dedifferentiated thyroid cancer.

## Molecular modifications of dedifferentiation

**Figure 1:**
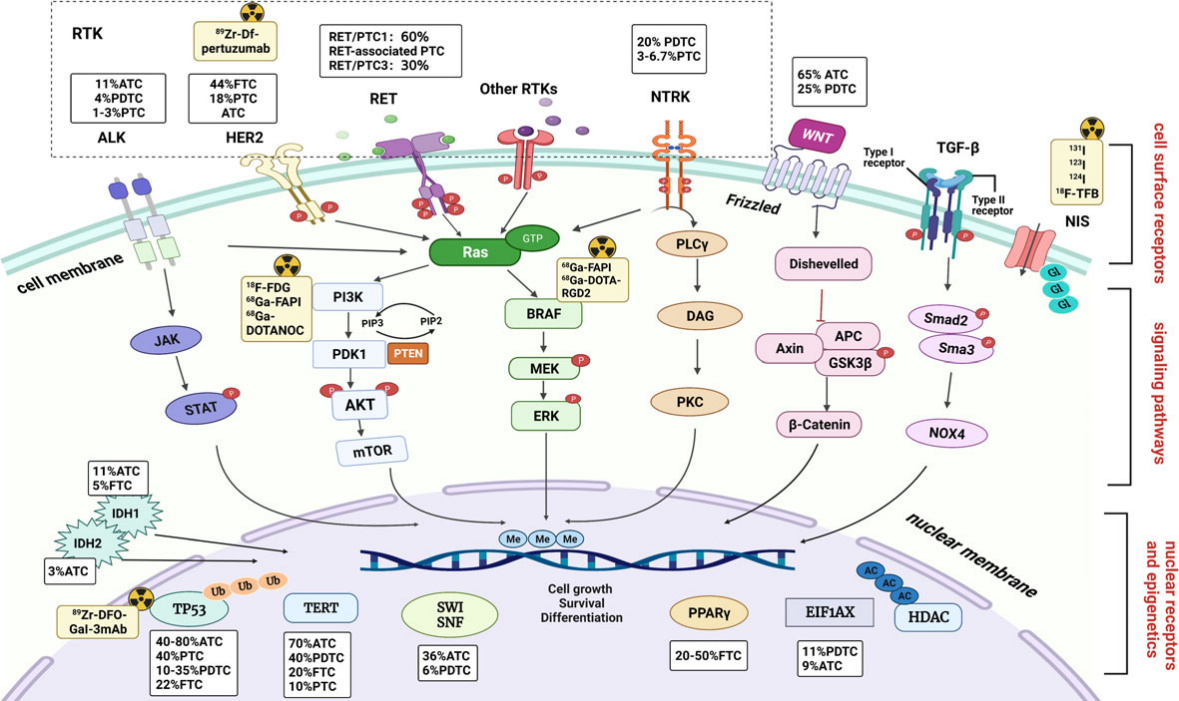
Mechanisms and molecular imaging involved in dedifferentiation of thyroid cancer. Molecular modifications and genetic mutations are described at the three levels of cell surface receptors, signaling pathways, nuclear receptors, and epigenetics. The incidence in different histologies are indicated in boxes. And the radioactive sign indicates the target for molecular imaging. RTK, receptor tyrosine kinase; ALK, anaplastic lymphoma kinase; HER2, human epidermal growth factor receptor 2; NTRK, neurotrophic tyrosine receptor kinase; NIS, sodium/iodide symporter; P, phosphorylation; JAK, Janus kinase; STAT, signal transducers and activators of transcription; ERK, extracellular signal-regulated kinase; PLCγ, phospholipase C-γ; DAG, diacylglycerol; PKC, protein kinase C; TGF-β, transforming growth factor-β; PI3K, phosphoinositide 3-kinase; APC, adenomatous polyposis coli; AXIN1, axis inhibition protein 1; GSK3β, glycogen synthase kinase 3β; NOX4, NADPH oxidase 4; PPAR-γ, peroxisome proliferator activated receptor gamma; HDAC, histone deacetylase; TERT, telomere reverse transcriptase; IDH, isocitrate dehydrogenase; EIF1AX, eukaryotic translation initiation factor 1A.

Dedifferentiated thyroid cancers lose their differentiation characteristics by various mechanisms, the most important of which is the decreased expression, localization, or abnormal function of sodium/iodide symporter (NIS) proteins (6, 7). Biomolecular modifications such as DNA methylation, protein phosphorylation, acetylation, ubiquitination, and glycosylation are significant epigenetic factors in thyroid cancer. The potential molecular basis for RAIR is the silencing of expression of thyroid-specific genes NIS, thyroglobulin (Tg), TSH receptor (TSHR), thyroperoxidase, transcription factors paired box gene-8 (PAX-8), and thyroid transcription factor-1, which are involved in alterations in cell surface receptors, signaling pathways, and nuclear receptors and epigenetics, respectively (4, 8) **(** **Figure 1****)**. The following detailed description is based on the site of molecular modifications and the expression levels.

### Cell surface receptors

Receptor tyrosine kinase (RTKs) is a transmembrane protein expressed in the cell membrane or adjacent to the plasm, which binds to specific ligands resulting in its autophosphorylation, and mutations in the gene could constitutively activate different downstream signaling pathways, ultimately leading to dysregulation of cell proliferation, dedifferentiation, and reduced apoptosis.

#### Neurotrophic tyrosine receptor kinase (NTRK)

NTRK genes include NTRK1, NTRK2, and NTRK3. The autophosphorylation of NTRK1 tyrosine residues in the tyrosine-kinase domain increase NTRK1 activity. NTRK gene fusions are oncogenic drivers that lead to NTRK gene fusions due to intra- or inter-chromosomal rearrangements, and cytoplasmic Trk fusion proteins activate downstream signals through phosphoinositide 3-kinase (PI3K), mitogen-activated protein kinase (MAPK), and phospholipase C-γ (PLCγ) to drive tumor proliferation and spread. NTRK fusions have been found in 3-6.7% of papillary thyroid cancer (PTC) and 20% of PDTC (9).

#### Anaplastic lymphoma kinase (ALK)

The ALK is a transmembrane tyrosine kinase of the insulin receptor family that, when the ligand binds to its extracellular structural domain, promotes activation of multiple downstream signaling pathways, such as PI3K/AKT, MAPK, and Janus kinase (JAK)-signal transducer and activator of transcription (STAT). ALK mutations and rearrangements are most common in ATC (11.1%) and PDTC (4%), and they play a role in disease progression and aggressiveness (10).

#### RET

The RET gene is a proto-oncogene that encodes the RET protein of the tyrosine kinase receptor superfamily. RET protein is a receptor tyrosine kinase, undergoing phosphorylation at several tyrosine residues, that can activate various downstream signaling pathways, such as MARK and PI3K, to induce cell proliferation. Rearrangements of the RET and other genes are common (5% - 25%) in PTC (3). The expression of thyroid-specific genes, increasing the differentiation process was suppressed by conditional activation of RET/PTC1 or RET/PTC3 (6). RET/PTC1 accounts for about 60% of RET-associated PTC, with RET/PTC3 accounting for approximately 30%. Although uncommon, RET/PTC rearrangements have been discovered in ATC and PDTC, primarily in carcinomas with a differentiated component.

#### Other RTKs

Copy number increases have been found in different subtypes of thyroid cancer such as epidermal growth factor receptor (EGFR), platelet-derived growth factor receptor A/B (PDGFRA/B), vascular endothelial growth factor receptor 1,2 (VEGFR1,2), mast/stem cell growth factor receptor kit (c-Kit) and metabotropic proto-oncogene receptor tyrosine kinase (MET). Missense mutations such as fibroblast growth factor receptor 2 (FGFR2) and FMS-like tyrosine kinase 3 (FLT3) were found in 11% and 17% of PDTC, respectively (3). The human epidermal growth factor receptor 2 (HER2) gene (ERBB2) overexpression was discovered in follicular thyroid cancer (FTC) (44%), PTC (18%), and some ATC (11). HER2 and HER3 are essential actors upstream of the signal-regulated kinase Extracellular Signal-Regulated Kinase (ERK) and AKT signaling pathways. Overexpression of HER2 and HER3 may provide a RAIR-DTC tumor escape mechanism for BRAF mutant cells treated with the BRAF inhibitor vemurafenib (12).

### Signaling pathway

#### PI3K pathway

##### RAS

The RAS proto-oncogene is one of the most common mutation targets in the PI3K/AKT cascade, and the G protein-like signaling protein it encodes is located on the inner surface of the cell membrane and is active when combined with GTP. Signaling pathways transmit signals from cell membrane RTKs and G protein-coupled receptors. The RasGRP3 mutation was shown to be more common in metastatic RAIR-DTC, promoting cell proliferation, invasion, and migration. RAS mutations, harboring more DNA hypermethylations, show the preferential association with AKT phosphorylation and are more likely to activate the PI3K/AKT pathway, which can occur in 30%-50% of FTC, 15% of PTC, 30%-45% of follicular variant papillary thyroid cancer (FVPTC), and 20%-40% of PDTC and ATC (13).

##### PIK3CA

Activating mutations or increases in the copy number of PIK3CA can increase the protein’s expression. Some studies have found that activation of PIK3CAE^545K^ plays a role in the progression of well-differentiated thyroid cancer to ATC. PIK3CA mutations are common in ATC (18%) but less common in PDTC (2%) and PTC (0.5%) (14).

##### AKT

The PI3K/AKT signaling pathway has long been recognized to regulate a variety of cellular and molecular processes, including cell growth, proliferation, and cell motility. AKT mutations represent a late event in thyroid cancer and, therefore, are more common in PDTC (19%) (15). In thyroid cancer cells, the phosphorylation of AKT reduced NIS and TSHR expression and RAI absorption. These findings imply that an activated AKT signaling pathway might be engaged in RAIR-DTC through mediating RasGRP3 mutation. Furthermore, suppression of the PI3K/AKT signaling pathway has been shown to promote NIS expression and RAI absorption in thyroid cancer cells (6).

##### PTEN

PTEN is a tumor suppressor gene that is found on chromosome 10 and is altered or deleted in heritable and spontaneous malignancies. PTEN is one of the most important downstream regulators of PI3K signaling, and its dysregulation could have a significant impact on this pathway. PTEN mutations, decreasing the conversion from PIP3 to PIP2 followed by increasing AKT phosphorylation, may prevent NIS from being glycosylated and inhibit it from reaching the plasma membrane. As a result, cytoplasmic NIS expression increases (16). PTEN mutations have been found in ATC (15%), FTC (14%), PDTC (4%), and PTC (2%) (3).

#### MAPK pathway

Many human cancer types exhibit activation of the MAPK signaling pathway, which is accomplished by activating mutations or overexpression of MAPK upstream activators such as RTKs, Ras, and Raf, leading to MEK phosphorylation followed by ERK phosphorylation. Changes in the MAPK pathway are prevalent in thyroid cancer, particularly in PTC (40–80%), ATC (10–50%), and PDTC (5–35%) (17). It mostly includes mutations in the BRAF gene, which result in cell differentiation loss and apoptosis inhibition. Furthermore, patients with BRAF mutations show hypermethylation of the TSHR gene promoter. The most frequent BRAF mutation is the V600E gene replacement, which boosts BRAF protein activity and keeps it active. It forms a monomer independent of the upstream RAS kinase, leading to persistent activation of MEK/ERK, cell differentiation loss, tumor development, and apoptosis inhibition. BRAF^V600E^ mutations are found in 45-50% of PTC and 36% of ATC (18).

#### WNT pathway

Molecular alterations in the Wnt/β-catenin signaling pathway involved in adenomatous polyposis coli (APC), axis inhibition protein 1 (AXIN1), and catenin beta 1 (CTNNB1) contribute to thyroid tumorigenesis. Furthermore, the direct phosphorylation of glycogen synthase kinase 3β (GSK3β) activates the WNT/β-catenin pathway and the association between β-direct catenin and PAX-8 boosts its gene transcription for NIS expression. These changes become more common in ATC (66%) and PDTC (25%) (19).

#### Transforming growth factor-β (TGF-β)/Smad signaling pathway

Several studies have found that TGF-β is essential for the proliferation and differentiation of thyroid cells (6). The TGF-β-type II receptor complex motivates the phosphorylation of Smad2 and Smad3. Some researchers demonstrated that a BRAF mutation could increase NADPH oxidase 4 (NOX4) expression in thyroid cancer cells by the TGFβ/SMAD3 signaling pathway. NOX4-reactive oxygen species (ROS) generation suppresses NIS expression in follicular cells by interfering with the binding of the PAX8 to the NIS gene promoter. NOX4 might be used as a therapeutic target in conjunction with other MAPK-kinase inhibitors to enhance their efficacy on RAIR-DTC redifferentiation (20).

### Nuclear receptors and epigenetic alterations

#### TP53

TP53 gene is an oncogene that encodes a protein involved in a variety of cellular activities, which could cause cell cycle arrest, apoptosis, senescence, DNA repair, or metabolic alterations in response to cellular stress. TP53 inactivation, its degradation mediated by p53 poly-ubiquitination, has long been thought to be a final step in tumor growth. TP53 mutations were found in around 40-80% of ATC, 10-35% of PDTC, 40% of PTC, and 22% of oncocytic FTC (21).

#### Telomere reverse transcriptase (TERT)

TERT maintains the length and stability of chromosomes by adding telomeres to the ends of chromosomes, which is of great significance to the lifespan of the body and various cellular activities. Mutations of the TERT promoter are mostly late events in the development of thyroid cancer, the incidence of which is high, especially in ATC (70%), PDTC (40%), FTC (20%), and PTC (10%) (15). TERT promoter mutations have also been shown to be strongly associated with aggressive clinicopathological characteristics and the probability of recurrence or distant metastasis (22).

#### SWI/SNF

The SWI/SNF complexes gene mutations have been detected in ATC (36%) and PDTC (6%) (3). SWI/SNF complexes are critical for maintaining differentiated function in thyroid cancer, and their loss imparts radioiodine refractoriness as well as resistance to MAPK inhibitor-based redifferentiation therapy (23).

#### Eukaryotic translation initiation factor 1A (EIF1AX)

The EIF1AX gene encodes an essential eukaryotic translation initiation factor. EIF1AX mutations have been reported in PDTC (11%) and ATC (9%) associated with oncogenic RAS. In advanced disease, the dramatic interplay of EIF1AX and RAS mutations shows that they may work together to induce tumor progression (24). The mechanism of EIF1AX mutation in thyroid cancer tumorigenesis and dedifferentiation still needs to be further studied.

#### Isocitrate dehydrogenase (IDH1/IDH2)

The IDH1 mutations are frequently found in thyroid cancer, identified in ATC (11%), FTC (5%), and PDTC (1.25%). While IDH2 mutation was identified in 3% of ATC (25). However, further research is needed to identify their functions in the pathogenesis of thyroid carcinomas.

#### Peroxisome proliferator activated receptor gamma (PPARγ)

PAX8/PPARγ rearrangement is the second most common genetic alteration in 20-50% of FTC besides RAS mutation, with an incidence of 30-35%, and it is also present in a minority of FVPTC (5%). It plays a role in the control of cell proliferation and redifferentiation. PPAR agonists have been proven to trigger redifferentiation in thyroid cancer in some studies (6).

#### Histone deacetylase (HDAC)

Notably, dysregulated histone acetyltransferase and HDAC activity are linked to cancer cell growth, proliferation, and differentiation. Some researchers have discovered that histone acetylation is altered in thyroid tumorigenesis and H3 histone is turned off in the progression from differentiated to undifferentiated thyroid cancer (26).

## Molecular imaging in detection of dedifferentiated thyroid cancer

Nowadays, molecular imaging, which uses radionuclides or intentionally changed molecules to find biomarkers, prospective therapy targets, or define signaling networks, has grown in popularity. These targets are important in the diagnosis and treatment of dedifferentiated thyroid cancer because they allow the molecular component of tumor tissue to be characterized and quantified. Molecular imaging has been demonstrated to help with thyroid cancer diagnosis, individualized treatment, and prognostic indicators prediction (27) **(** **Figure 1****)**. Furthermore, molecular imaging is required for multimodality-based thyroid cancer treatment options, which could drive the invention of novel therapeutic or diagnostic tracers (28). Recently, a growing number of clinical studies have explored molecular imaging in dedifferentiated thyroid cancer.

### Sodium iodide symporter targeted molecular imaging (NIS)

#### Radioiodine

A widely used radioisotope, radioiodine, plays a critical role in the diagnosis and treatment of DTC, such as ^123^I, ^124^I, and ^131^I. ^131^I SPECT/CT has become a routine tool for visualizing the lesions and evaluating distant metastases in patients receiving radioactive iodine therapy (29). ^124^I PET/CT could improve the sensitivity and spatial resolution of SPECT/CT, leading to superior diagnostic performance of post-therapy 131I-WBS. However, it is expensive and has low accessibility (30) (**Table 1**).

**Table 1:** The diagnostic efficacy of radiotracers in dedifferentiated thyroid cancer with negative post-therapy ^131^I-WBS and elevated Tg

Radiotracers	Study Phase	Population	n	Sensitivity	Specificity	Accuracy	PPV	NPV
^124^I	Prospective (2016) (30)	DTC	17	44%	100%	NA	100%	62%
^18^F-TFB	Retrospective (2020) (32)	recurrent DTC	25	64%	NA	64%	100%	NA
^18^F-FDG	Retrospective (2021) (34)	DTC	113	92%	94%	93%	87%	93%
^68^Ga-DOTANOC	Prospective (2019) (36)	DTC	62	78.4%	100%	82.3%	100%	50%
^68^Ga-DOTA-RGD_2_	Prospective (2020) (37)	RAIR-DTC	44	82.3%	100%	82.4%	NA	NA
^68^Ga-PSMA	Retrospective (2020) (39)	RAIR-DTC	5	NA	NA	NA	NA	NA
^68^Ga–FAPI	Prospective (2022) (40)	metastatic DTC	35	83% in neck lesions, 79% in distant metastases	NA	NA	NA	NA

RAIR, radioactive iodine-refractory; DTC, differentiated thyroid cancer; Tg, thyroglobulin; n, number; NA, not available; PPV, positive prognostic value; NPV, negative prognostic value; ^18^F-TFB, fluorine-18-tetrafluoroborate; ^18^F-FDG, fluorine-18-fluorodeoxyglucose; PSMA, prostate-specific membrane antigen; FAPI, fibroblast activation protein inhibitor.

#### Fluorine-18-tetrafluoroborate (^18^F-TFB)

TFB is a sodium/iodide symporter substrate with similar NIS affinities to radioiodine. ^18^F-TFB has recently been established as a flexible PET probe for imaging the activity of human sodium/iodide symporters. As a result, ^18^F-TFB PET could be a valuable method for evaluating NIS expression in human diseases and be able to visualize DTC metastases in negative ^124^I PET (31). Compared to conventional diagnostic WBS and SPECT-CT, ^18^F-TFB PET could detect more local recurrence or metastases of DTC (32). The combination of ^18^F-TFB PET and fluorine-18-fluorodeoxyglucose (^18^F-FDG) PET appears to be a feasible technique for characterizing DTC tumor presentations in terms of differentiation and, as a result, individually planning and monitoring therapy. Prospective studies evaluating the potential of ^18^F-TFB PET in recurrent DTC are needed in the future.

### Glucose transporter targeted molecular imaging

#### ^18^F-FDG

^18^F-FDG is well-known radiopharmaceutical glucose that is mostly carried by glucose-transporter family-1 (GLUT1), and its uptake has been reported to be influenced by the degree of tumor proliferation and differentiation. Additionally, the surface expression of GLUT is controlled by the PI3k/AKT pathway. Advanced TC with low radioiodine uptake usually had high ^18^F-FDG uptake. Some researchers observed that ^18^F-FDG showed positive uptake in 50 patients (17%) among 258 DTC patients, 39 (78%) of which did not show positive lesions on post-therapy WBS (33). ^18^F-FDG PET/CT might allow RR-DTC patients to classify their prognosis by revealing tumor aggressiveness. ^18^F-FDG PET/CT has shown good diagnostic performance in non-iodine avid DTC with a sensitivity, specificity, and accuracy of 92%, 94%, and 93%, respectively. Therefore, ^18^FDG PET/CT could enable clinicians in identifying individuals with RAIR-DTC and developing a treatment strategy earlier (34).

### Peptide-based molecular imaging

#### Somatostatin receptor (SSTR)

SSTRs are highly expressed in neuroendocrine tumors. But in recent studies, SSTRs have been found to be overexpressed in dedifferentiated thyroid cancer. Less differentiated carcinomas are more likely to express a wider range of SSTR subtypes, primarily subtypes 2, 3, and 5, bolstering the theories of peptide receptor-based nuclear diagnosis and treatment. SSTR1-5 activation suppresses PI3K/AKT signaling (35). Parveen et al. evaluate the value of ^68^Ga-DOTANOC PET/CT in DTC with negative ^131^I WBS and elevated serum Tg levels. The detection of recurrent disease in DTC with a sensitivity and specificity of 78.4%, and 100%, respectively. It may also assist in the selection of possible peptide receptor radionuclide treatment candidates (36).

#### αvβ3 Integrin

The integrin αvβ3 is overexpressed in the tumor vascular system. The tripeptide sequence arginine-glycine-aspartate (RGD) shows a high affinity and specificity for integrin αvβ3. Recently, a prospective study has indicated that ^68^Ga-DOTA-RGD_2_ PET/CT showed a better diagnostic performance in RAIR-DTC with negative post-therapy ^131^I-WBS with an accuracy, sensitivity, and specificity of 86.4%, 82.3%, and 100%, respectively, compared to ^18^F-FDG PET/CT [75%, 82.3%, 50% (37)]. Moreover, the novel radiotracer could provide the potential for the selection of eligible RAIR-DTC candidates for treatment with ^177^Lu-DOTA-RGD_2_.

#### Prostate-specific membrane antigen (PSMA)

PSMA is a type II transmembrane glycoprotein receptor expressed in prostate cancer cells and the endothelium of tumor-associated neovasculature in several malignancies. Similarly, PSMA expression has been observed in 62% of persistent or recurrent DTC. Some studies have shown that PSMA expression was also related to poor prognosis and that very high PSMA expression was associated with poorer PFS (38). For patients with RAIR-DTC, ^68^Ga-PSMA PET/CT can be useful for staging because it could identify different types of lesions and may discover lesions that ^18^FDG PET/CT does not detect. Additionally, ^68^Ga-PSMA might be utilized to screen patients for ^177^Lu-PSMA targeted therapy in the future (39).

### Other molecular imaging

#### ^68^Ga–labeled fibroblast activation protein inhibitor (FAPI)

In over 90% of epithelial carcinomas, FAP is significantly expressed in cancer-associated fibroblasts. Increased FAP expression is associated with dedifferentiation and aggressiveness outcome of thyroid cancer. In some cases, ^68^Ga–FAPI PET/CT revealed high activity in the metastatic DTC with elevated Tg and negative iodine scan. ^68^Ga-FAPI might perform better than ^18^F-FDG in detecting metastatic DTC, particularly in pulmonary and lymph node metastases (40). Another research has also found that ^68^Ga-DOTA-FAPI-04 PET/CT may have a good performance in the detection of lymph node metastasis and distant metastasis in 87.5% (21/24) of RAIR-DTC patients (41). More multicenter prospective studies with bigger sample sizes are needed to confirm these findings.

#### Lectin galactoside-binding soluble 3

Galectin-3 (Gal-3) is a β-galactoside binding protein of the lectin family that is absent in normal and benign thyroid tissues but overexpressed in the cytoplasm, cell membranes, and intercellular components of DTC and ATC (42). Meanwhile, Gal-3 is a physiological target of p53 transcriptional activity, and its downregulation mediated by p53 is essential for p53-induced apoptosis. ^89^Zr-DFO-GaI-3mAb detected specific and reliable uptake of human thyroid cancer xenograft *in vivo*. ^89^Zr-DFO-F(ab’)2 anti-gal-3 exhibited specific uptake in tumor tissue, while the normal thyroid tissue had no uptake. Besides, in the absence of radioiodine uptake, specific and selective detection of thyroid tumors was achieved by targeting Gal-3 (43). Gal-3 immunoPET is still a new field of research, and these findings imply that diagnostic and clinical applications of Gal-3 targeted radiotracers for thyroid cancer need further investigation.

#### HER2

In a recent study, a HER2-specific PET imaging probe ^89^Zr-Df-pertuzumab was developed to assess the diagnostic effectiveness in orthotopic ATC. These findings suggested that noninvasive HER2 molecular imaging offers a great potential for detecting HER2 status in ATC (11). With extensive clinical translation and use of ^89^Zr-Df-pertuzumab PET, this imaging method may be able to identify the diverse levels of HER2 around the body. This suggests that this unique imaging method could identify ATC patients who may react to HER2-targeted therapy (such as pertuzumab and trastuzumab) and dynamically monitor therapeutic responses.

## Landscape of treatment in dedifferentiated thyroid cancer

### Tyrosine kinase inhibitors (TKIs)

In the past decade, the findings of signaling pathways and activating mutations have spurred the development of biomarker-driven targeted therapies. Most extensively investigated and clinically approved targeted therapies in thyroid cancer include the TKIs that target antiangiogenic markers, BRAF mutation, and MAPK pathway components. The initiation into systemic treatment is based on tumor burden and tumor growth rate. Watchful surveillance can be considered in patients with stable or slowly progressive thyroid cancer. Patients with rapidly progressive and/or symptomatic diseases are candidates for TKIs (44).

#### Multi-kinase inhibitors (MKIs)

MKIs inhibit the activity of multiple receptor tyrosine kinases such as VEGFR, PDGFR, FGFR, and various Raf kinases, thereby suppressing tumor cell proliferation and angiogenesis. Novel MKIs have been evaluated and approved by FDA for advanced RAIR-DTC such as sorafenib, lenvatinib and cabozantinib (45–47). Other commercially available MKIs (such as anlotinib, donafenib, surufatinib, sunitinib, and pazopanib) can be considered if clinical trials are not available or appropriate **(** **Tables 2****,** **3****)** (48–52). MKIs have demonstrated clinical efficacy to prolong median progression-free survival (PFS), but in most cases, no significant benefit was observed in overall survival (OS), except in the SELECT study of lenvatinib, OS was significantly improved among patients aged > 65 years compared with placebo (53). Due to these multiple target effects, molecular testing does not predict clinical responses. And the off-target side effects are common and sometimes severe. The most common treatment-related adverse events (TRAEs) include diarrhoea, fatigue, hypertension, hand-foot skin reactions et al. Most adverse effects can be managed and are reversible with discontinuation. Below, we summarize the most important results of TKIs clinical trials in advanced or dedifferentiated thyroid cancer.

**Table 2:** Published pivot clinical trials for RAIR DTC and ATC.

Agents	Targets	Phase	Clinical Trials	population	n	PFS (month)	OS (month)	ORR	Dosage	Common TRAEs
Sorafenib (2014) (45)	VEGFR1-3, PDGFR, RET, RAF, c-KIT	III	NCT00984282 (DECISION)	RAIR-DTC	417	10.8 vs. 5.8 of placebo arm	Not reached	12.2% vs. 0.5%	400 mg orally twice daily	hand-foot skin reaction, diarrhoea, alopecia
Sorafenib (2013) (87)	VEGFR1-3, PDGFR, RET, RAF, c-KIT	II	NCT00126568	ATC	20	1.9	3.9	10%	400 mg orally twice daily	fatigue, anemia, hypocalcemia
Lenvatinib (2015) (46)	VEGFR1-3, PDGFR, RET, FGFR I-4, c-KIT	III	NCT01321554 (SELECT)	RAIR-DTC	392	18.3 vs. 3.6 of placebo arm	Not reached	64.8% vs. 1.5%	24 mg orally once daily	hypertension, diarrhoea, fatigue
Lenvatinib (2017) (88)	VEGFR1-3, PDGFR, RET, FGFR I-4, c-KIT	II	NCT01728623	ATC	17	7.4	10.6	24%	24 mg orally once daily	decreased appetite, hypertension, fatigue, nausea, proteinuria
Lenvatinib (2021) (89)	VEGFR1-3, PDGFR, RET, FGFR I-4, c-KIT	II	NCT02657369	ATC	34	2.6	3.2	2.9%	24 mg orally once daily	hypertension, decreased appetite, fatigue, and stomatitis
Cabozantinib (2021) (47)	Tie-2, c-MET, KIT, VEGFR1, VEGFR2, RET	III	NCT03690388 (COSMIC-311)	RAIR-DTC	227	Not reached vs. 1.9 of placebo arm	Not reached	15% vs. 0	60 mg orally once daily	hand-foot syndrome, diarrhoea, nausea
Anlotinib (2020) (48)	VEGFR, PDGFR, FGFR, and c-Kit	II	NCT02586337	RAIR-DTC	113	40.5 vs.8.4 of placebo arm	Not reached	59.2% vs. 0	12 mg orally once daily	hypertension, hypertriglyceridemia
Donafenib (2021) (49)	VEGF, PDGF, RAF	II	NCT02870569	RAIR-DTC	35	14.98 in 300 mg arm and 9.44 months in 200 mg arm	NA	13.3% in 300 mg arm and 12.5% in 200 mg arm	200 mg/300 mg orally twice daily	palmar-plantar erythrodysesthesia and hypertension
Surufatinib (2020) (50)	VEGFR, FGFR	II	NCT02588170	RAIR-DTC, MTC	59	11.1	NA	23.2%	300 mg orally once daily	hypertension, proteinuria, elevated blood pressure, hypertriglyceridemi, pulmonary inflammation
Sunitinib (2017) (51)	PDGFR, FLT3, c-KIT, VEGFR, RET	II	NCT00510640	RAIR-DTC/ATC	41/4	13.1/NA	26.4/NA	22%/0%	50 mg orally once daily	asthenia/fatigue, mucosal cutaneous toxicities, hand-foot syndrome
Pazopanib (2010) (52)	VEGF, PDGFR, c-kit	II	NCT00625846	RAIR-DTC	37	NA	NA	49%	800 mg orally once daily	atigue, hair hypopigmentation, diarrhoea, nausea
Apatinib (2022) (59)	VEGFR-2	III	NCT03048877 (REALITY)	RAIR-DTC	92	22.2 vs. 4.5 of placebo arm	Not reached vs.29.9	54.3% vs. 2.2%	500 mg orally once daily	hypertension, hand-foot syndrome, proteinuria
Axitinib (2014) (60)	VEGF, PDGFR, c-kit	II	NCT00094055	advanced thyroid cancer of any histology	60	15	35	38%	5 mg orally twice daily	hypertension, proteinuria, diarrhea, weight decrease
Vemurafenib (2016) (62)	BRAF	II	NCT01286753	RAIR-DTC (BRAF^V600E^+)	51	18.2 in TKI-naïve cohort; 8.9 in non- TKI-naïve cohort	Not reached	38.50% in TKI-naïve cohort; 27.3% in non- TKI-naïve cohort	960 mg orally twice daily	rash, fatigue, asthenia, alopecia
Dabrafenib + Trametinib (2018) (66)	BRAF^V600E^, MEK	II	NCT02034110	RAIR-DTC (BRAF^V600E^+)	16	Not reached	Not reached	66%	Dabrafenib 150 mg orally twice daily + Trametinib 2mg orally once daily	fatigue, pyrexia, nausea
Selumetinib +RAI (2022) (68)	MEK 1/2	III	NCT01843062 (ASTRA)	DTC at high risk of primary treatment failure	233	NA	NA	CR rate for selumetinib+ RAI (40%) vs. placebo+RAI (38%)	75 mg orally twice daily	rash, fatigue, diarrhea, peripheral edema
Larotrectinib (2018) (71)	NTRK1/2/3, ROS1, ALK	I/II	NCT02122913 NCT02637687 NCT02576431	TRK fusion (+) solid tumor	55 (5 thyroid cases)	Not reached	NA	75%	100 mg orally twice daily	increased ALT or AST leve, fatigue, vomiting
Entrectinib (2020) (73)	NTRK1/2/3, ROS1, ALK	I/II	NCT02650401 NCT02097810 NCT02568267	NTRK fusion (+) solid tumor including TC	54	NA	NA	57%	600 mg orally once daily	increased weight, anaemia
Everolimus (2018) (75)	mTOR	II	NA	RAIR-DTC/ATC	33/7	12.9/NA	Not reached/NA	3%/14.3%	10 mg orally once daily	mucositis, acneiform rash, fatigue, cough
Temsirolimus + Sorafenib (2017) (77)	mTOR + VEGFR, PDGFR, BRAF	II	NCT01025453	RAIR-DTC	36	NA	NA	22.0%	sorafenib 200 mg orally twice a day and temsirolimus 25 mg intravenous weekly	hyperglycemia, fatigue, anemia, and oral mucositis
Pralsetinib (2021) (79)	RET	I/II	NCT03037385 (ARROW)	RET fusion (+) thyoird caner	20	Not reached	Not reached	89%	400 mg orally once daily	hypertension, neutropenia, lymphopenia,

RAIR, radioactive iodine-refractory; DTC, differentiated thyroid cancer; ATC, anaplastic thyroid cancer; n, number; NA, not available; PFS, progression-free survival; OS, overall survival; ORR, objective response rate; TRAEs, treatment-related adverse events. VEGFR, vascular endothelial growth factor receptor; PDGFR, platelet-derived growth factor receptor; EGFR, epidermal growth factor receptor; FGFR, fibroblast growth factor receptor; c-kit, mast/stem cell growth factor receptor kit, NTRK, neurotrophic tyrosine receptor kinase; FLT3, FMS-like tyrosine kinase 3; ALK, anaplastic lymphoma kinase.

**Table 3:** Ongoing TKIs clinical trials for RAIR-DTC and ATC.

Drugs	Mechanism/Targets	Clinical Trials	Population	Phase	Status
Anlotinib	VEGFR, PDGFR, FGFR, and c-Kit	NCT02586337	DTC	III	Terminated
Donafenib	VEGF, PDGF, RAF	NCT03602495 (DIRECTION)	RAIR DTC	III	Terminated
Vandetanib	RET, VEGFR, EGFR	NCT01876784 (VERIFY)	locally advanced or metastatic DTC	III	Active, not recruiting
Lenvatinib, Denosumab	VEGFR1-3, PDGFβ, RET, FGFR-I +RANKL(Bone metastases from RAI-R DTC)	NCT03732495 (LENVOS)	Bone Metastatic RAIR DTC	II	Recruiting
Dabrafenib, Trametinib	BRAF^V600E^,^K601E^ + MEK	NCT03244956 (MERAIODE)	Metastatic RAIR TC	II	Active, not recruiting
Dabrafenib, Trametinib	BRAF^V600E^,^K601E^ + MEK	NCT04940052	BRAF^V600E^(+) RAIR-DTC with previous treatment	III	Recruiting
Dabrafenib, Trametinib(Neoadjuvant)	BRAF^V600E^,^K601E^ + MEK	NCT04739566 (ANAPLAST-NEO)	ATC	II	Recruiting
Dabrafenib, Lapatinib	BRAF ^V600E^, ^K601E^ + EGFR, HER	NCT01947023	RAIR TC	I	Active, not recruiting
Selpercatinib (Neoadjuvant)	RET	NCT04759911	RET-altered thyroid cancer	II	Recruiting
Crizotinib	ALK, ROS1	NCT02465060	Solid cancer	II	Recruiting
Sorafenib, Everolimus	VEGFR, PDGFR, BRAF + mTOR	NCT02143726	Advanced, RAIR Hurthle Cell Thyroid Cancer	II	Active, not recruiting

RAIR, radioactive iodine refractory; DTC, differentiated thyroid cancer; TC, thyroid cancer; PTC, papillary thyroid cancer; ATC, anaplastic thyroid cancer; VEGFR, vascular endothelial growth factor receptor; PDGFR, platelet-derived growth factor receptor; EGFR, epidermal growth factor receptor; FGFR, fibroblast growth factor receptor; PD-L1, programmed death-ligand 1; c-kit, mast/stem cell growth factor receptor kit; HER, human epidermal growth factor receptor; RANKL, receptor activator for nuclear factor-κ B ligand.

##### Sorafenib

Sorafenib is an orally active TKI that targets VEGFR, RET, RAF, and PDGFR et al. In the DECISION trial, sorafenib showed significantly longer PFS (10.8 months vs. 5.8 months in the placebo arm) and a 12% objective response rate (ORR) (45). Sorafenib was approved by U.S. Food and Drug Administration (FDA) in 2013 as the first TKI for RAIR-DTC. In subsequent clinical practice, cases are reported and reveal tumor shrinkage efficacy of sorafenib as neoadjuvant treatment for unresectable thyroid carcinoma (54).

##### Lenvatinib

Lenvatinib, an orally active multi-targeted TKI, has been approved by both the FDA and the European Medicines Agency (EMA) for advanced and progressive RAIR-DTC. The phase III SELECT trial demonstrated significant improvements in median PFS (18.3 months vs 3.6 months) and ORR (64.8% vs. 1.5%) compared lenvatinib to placebo in 392 RAIR-DTC with or without previous TKI (46). Of note, OS was significantly improved among patients aged > 65 years (53). Lenvatinib may have a neoadjuvant role in selected cases of locally advanced DTC to reduce tumor volume and facilitate complete resection (55, 56). A real-world study demonstrated that treatment with first-line lenvatinib followed by another second-line therapy, including other TKI such as sorafenib or cabozantinib/chemotherapy/immunotherapy, may deliver a clinical benefit for RAIR-DTC. This study added evidence to a sequential strategy for the treatment of RAIR-DTC (57).

##### Cabozantinib

Cabozantinib is a selective inhibitor of MET, VEGFR-2, and RET et al. In the COSMIC-311 phase III trial for RAIR-DTC patients who failed first-line therapy with sorafenib and/or lenvatinib, cabozantinib showed significant improvement in PFS over placebo (median PFS not reached vs. 1.9 months) and in ORR (15% vs. 0%). Based on the COSMIC-311 study, FDA approved cabozantinib for advanced thyroid cancer as second-line therapy in September 2021 (47).

##### Anlotinib

Anlotinib is a novel multitarget tyrosine kinase inhibitor targeting VEGFR, PDGFR and FGFR et al. Outcome from a phase II trial of anlotinib vs. placebo for RAIR-DTC showed promising clinical efficacy with a prolonged median PFS (40.5 months vs. 8.3 months) and ORR of 59.2% (48). Notably, all of the enrolled patients were TKI-naive, which may contribute partially to the extraordinary clinical efficacy. Phase III study of anlotinib for RAIR-DTC has been completed and the trial data will be published soon. Based on its promising efficacy, anlotinib is currently approved by the Chinese National Medical Products Administration (NMPA) for the indication of RAIR-DTC. A report in 2021 ASCO showed that 10 out of 13 (76.9%) locally advanced thyroid cancer patients achieved partial response (PR), reflecting a significant prospect of anlotinib for neoadjuvant therapy in unresectable RAIR-DTC.

#### Donafenib

Donafenib, a modified form of sorafenib with a trideuterated N-methyl group, inhibits VEGFR, PDGFR, and various Raf kinases with improved molecular stability and pharmacokinetic profile (58). In the phase II dose exploratory study of donafenib for RAIR-DTC, the 300 mg arm showed clinical benefit in terms of PFS (14.98months) and ORR (13.3%) as well as tolerable safety profile (49). Phase III clinical trial to assess donafenib vs. placebo among patients with RAIR-DTC has been completed and is expected to unveil soon.

#### Anti-angiogenic agents

##### Apatinib

Apatinib is a selective VEGFR-2 inhibitor with potent anti-angiogenic activity. In a most recent REALITY phase III trial for RAIR-DTC (n=92) (59), apatinib showed promising efficacy over placebo in median PFS (22.2 months vs. 4.5 months) and ORR (54.3% vs. 2.2%). It is worth noting that apatinib also showed significant clinical benefits in OS (not reached vs. 29.9 months).

##### Axitinib

Another selective inhibitor of VEGF to block angiogenesis is axitinib. In a phase II trial (n=60) (60), axitinib appears active and well-tolerated in RAIR-DTC of any histology with ORR of 38% and median PFS of 15 months. Another study evaluated the comparative efficacy of axitinib as first-line or second-line treatment options. More favorable efficacy was observed in first-line treatment with an ORR of 53% and a median PFS of 13.6 months, while the counterparts in second-line treatment descended to 16.7% and 10.6 months, which might be ascribed to anti-angiogenic cross-resistance (61). More studies are warranted to explore the mechanism of TKI resistance and schedules of sequential treatment for RAIR-DTC.

#### MAPK signaling pathway inhibitors

As mentioned above, MKIs do not target specific mutations, which may compromise the safety and durability. Screening molecular abnormalities and practicing genotype-tailored agent selection may boost both anti-tumor efficacy and improve safety profile. Specifically, kinase inhibitors targeted BRAF^V600E^ and MEK have been studied in advanced thyroid cancer (62–66).

##### Vemurafenib

Vemurafenib is an oncogenic BRAF kinase inhibitor that has been approved for BRAF-positive melanoma. In a phase II study of vemurafenib for advanced thyroid cancer (n=51), the PR rate is 38.5% in the TKI-naive cohort (n=26) and 27.3% in the cohort with previous TKIs (n=25) (62). Vemurafenib also showed the ability to restore RAI avidity in BRAF mutant RAIR-DTC patients with 4 out 10 patients responding to radioactive iodine (63).

##### Dabrafenib and trametinib

Dabrafenib is a BRAF inhibitor and trametinib is a MEK inhibitor. In two preliminary trials for advanced thyroid cancer patients harboring BRAF^V600E^mutation, dabrafenib demonstrated clinical efficacy with PR of 30.1% (n=13) and the ability of RAI resensitization (6 out of 10 patients) (64, 65). Another landmark phase II trial enrolled 16 BRAF^V600E^positive ATC patients receiving the combination of dabrafenib and trametinib. The ORR was 69% and the estimated 12-month OS was 80% (66). Based on this study, the combination of dabrafenib and trametinib was approved by FDA for ATC with BRAF^V600E^ mutation in 2018.

##### Selumetinib

Selumetinib is another potent selective inhibitor of MEK1/2. In a phase II ‘proof of concept’ trial, selumetinib showed the ability to reverse refractoriness to radioiodine in patients with metastatic thyroid cancer, especially in RAS-mutant disease (67). However, in the phase III ASTRA trial, selumetinib plus adjuvant RAI failed to improve complete remission (CR) rates in patients with DTC at high risk of primary treatment failure versus RAI alone (68).

#### ALK inhibitor

ALK is a kinase that activates MAPK and PI3K/AKT pathways and is associated with younger age and aggressive behavior in DTC. As reported, ATC patients with ALK rearrangement responded well to ALK inhibitor crizotinib (69, 70). But the experience of the ALK inhibitor in advanced thyroid cancer is still limited and studies are needed to evaluate the efficacy and safety profile of ALK-dependent advanced thyroid cancer.

#### NTRK inhibitors

Though rarely thyroid cancers can be driven by rearrangements of the NTRK gene, selective inhibitors of TRK kinases larotrectinib or entrectinib provide clinical efficacy in patients with thyroid cancer harboring mutations or rearrangements in the NTRK genes (71–73). In a phase I/II trial, larotrectinib proved to be highly potent with 75% ORR for tumors harboring TPK-fusions including thyroid carcinoma (71). In another phase I/II trial for patients with NTRK fusion-positive solid tumors (n=54), entrectinib resulted in a favorable outcome with an ORR of 57%, including 4 (7%) of CR and 27 (50%) of PR (73). The promising efficacy and safety profile highlight NTRK inhibitors as an optional treatment for selective advanced thyroid cancer though more investigations are warranted.

#### PI3K/AKT/mTOR signaling pathway inhibitors

Dysregulation of the PI3K pathway has been implicated in oncogenesis and tumor progression, however, buparlisib, a pan-PI3K inhibitor, failed to show the benefit of PFS in RAIR FTC and PDTC (74). As for the inhibitors of downstream mTOR, studies showed that PI3K/mTOR/Akt-mutated dedifferentiated thyroid cancer patients appeared to benefit from mTOR inhibitors, such as everolimus, sirolimus and temsirolimus (75, 76). However, given the relatively low ORR observed, the mTOR inhibitors have not been clinically used as a single agent in the treatment of advanced thyroid cancer. Notably, given that inhibition of mTORC1 may lead to MAPK pathway activation through a PI3K-dependent feedback loop, the potential of a combined therapeutic approach with mTOR and MAPK inhibitors may be underscored. In a phase II trial, 36 metastatic RAIR-DTC received treatment with the combination of sorafenib and temsirolimus. PR was observed in 8 patients (22%), SD in 21 (58%), and PD in 1 (3%); patients who received no prior systemic treatment had a better response rate (77).

#### RET alteration inhibitors

Multikinase inhibitors with RET inhibitor activity, such as cabozantinib and vandetanib, have been evaluated for tumors with activating RET gene alterations including thyroid cancer, mainly in MTC. However, due to the nonselective nature of multikinase inhibitors, the safety and durability of responses to these agents are at least partially limited by off-target toxic effects. Noval generation of high selective RET alteration inhibitors, pralsetinib and selpercatinib demonstrated promising efficacy and favorable safety profile, and have been approved by FDA for RET-mutant medullary thyroid cancer and RET fusion-positive thyroid cancer (78–80). Though RET mutations occur mainly in medullary thyroid cancers and RET fusions occur rarely in follicular-derived thyroid cancers, novel RET alteration inhibitors may also alter the landscape of RET-dependent advanced thyroid cancers.

### HDAC inhibitors

HDAC seems to play a role in regulating the transcription of genes involved in ATC. HDAC inhibitors (HDACIs) can induce tumor growth arrest, differentiation, and apoptosis, and sensitize tumor cells to radiation, increase radioiodine uptake and intratumoral radioiodine accumulation (81). In preclinical models, HDACIs represent anti-tumor activity and the ability to restore RAI-avidity both as monotherapy and in combination with other anticancer agents (82). However, in two clinical trials, valproic acid, a HDAC inhibitor, failed to show anticancer activity in RAIR-DTC or ATC (83, 84).

### Targeted therapy in ATC

According to heterogeneous mutation and heavy mutant burden, ATC remains intractable to existing treatments. Several novel therapeutic approaches have been proposed in ATC. As mentioned above, dabrafenib and trametinib, BRAF and MEK inhibitors, have been approved by FDA for BRAF^V600E^-mutated ATC patients (66). The combination of dabrafenib and trametinib is also used as a novel neoadjuvant attempt for patients with initially unresectable BRAF^V600E^-mutated ATC (85, 86). As for other MKIs, sorafenib exhibited modest efficacy with a PR rate of 10%, SD rate of 25% and median PFS of 1.9 months in patients with ATC (n=20) (87). Lenvatinib demonstrated clinical activity in ATC patients (n=17) with a median PFS of 7.4 months, median OS of 10.6 months, and ORR of 24% (88). But in a most recent study for ATC (n=34), lenvatinib showed limited efficacy with ORR of 2.9%, PFS of 2.6 months, and OS of 3.2 months (89). Taken together, monotherapy of TKI may be not potent enough for ATC, and more investigations are needed to evaluate TKIs in combination with other novel agents such as anti-PD-1/L1 antibodies for the treatment of ATC **(** **Table 4****)**.

**Table 4:** Clinical trials of ICIs for RAIR-DTC and ATC.

Modality	Combination Type	Targets	Clinical Trials	Population	Phase	Status
Pembrolizumab (2019) (102)	ICI	PD-1	NCT02054806	Advanced solid tumors	I	Completed
Pembrolizumab (2020) (103)	ICI	PD-1	NCT02628067	Advanced solid tumors	II	Recruiting
Pembrolizumab	ICI	PD-1	NCT02688608	ATC, PDTC	II	Completed
Pembrolizumab	ICI	PD-1	NCT03012620	Rare cancers	II	Active, not recruiting
Spartalizumab(2020) (106)	ICI	PD-1	NCT02404441	Advanced solid tumors	I/II	Completed
Durvalumab	ICI	PD-L1	NCT03215095	Recurrent/Metastatic TC	I	Active, not recruiting
Nivolumab, Ipilimumab	ICI+ICI	PD-1, CTLA-4	NCT03246958	RAIR DTC, ATC	II	Active, not recruiting
Nivolumab Ipilimumab	ICI+ICI	PD-1, CTLA-4	NCT02834013	Rare tumors	II	Recruiting
Durvalumab, Tremelimumab	ICI+ICI	PD-L1, CTLA-4	NCT03753919 (DUTHY)	DTC, ATC	II	Recruiting
Pembrolizumab, Lenvatinib	ICI+TKI	PD-1,VEGFR1-3, PDGFR, RET, FGFR I-4,c-KIT	NCT02973997	RAIR DTC	II	Active, not recruiting
Pembrolizumab, Lenvatinib	ICI+TKI	PD-1,VEGFR1-3, PDGFR, RET, FGFR I-4,c-KIT	NCT04171622	ATC	II	Not yet recruiting
Nivolumab, Encorafenib/Binimetinib	ICI+TKI	PD-1	NCT04061980	RAIR BRAF-mutated DTC	II	Recruiting
Atezolizumab, Cabozantinib	ICI+TKI	PD-L1,Tie-2, c-MET, KIT, VEGFR1, VEGFR2, RET	NCT03170960	Locally advanced or metastatic solid tumors	Ib	Active, not recruiting
Atezolizumab, Cabozantinib	ICI+TKI	PD-L1Tie-2, c-MET, KIT, VEGFR1, VEGFR2, RET	NCT04400474	endocrinal tumors	II	Recruiting
Avelumab, Regorafenib	ICI+TKI	PD-L1,VEGFR 1-3, KIT, PDGFR-α, PDGFR-β, RET	NCT03475953	RAIR DTC	I/II	Recruiting
Cemiplimab, Dabrafenib, Trametinib	ICI+TKI+TKI	PD-1, BRAF^V600E^ mutation	NCT04238624	ATC (BRAF^V600E^+)	II	Recruiting
Pembrolizumab, Dabrafenib, Trametinib (neoadjuvant)	ICI+TKI+TKI	PD-1, BRAF, MEK	NCT04675710	ATC	II	Recruiting
Nivolumab, Ipilimumab, Cabozantinib	ICI+ICI+TKI	PD-1 andCTLA-4,Tie-2, c-MET, KIT,VEGFR1, VEGFR2, RET	NCT03914300	Advanced DTC	II	Active, not recruiting
Pembrolizumab, Docetaxel	ICI+CT	PD-1	NCT03360890	TC and salivary gland tumors	I	Recruiting
Pembrolizumab, Docetaxel, Doxorubicin	ICI+CT	PD-1	NCT03211117	ATC	II	Completed
Atezolizumab, Vemurafenib/Cobimetinib//Bevacizumab/Paclitaxel	ICI+TKI/anti-angiogenesis agents/CT	PD-L1, BRAF^V600E^/MEK/VEGF	NCT03181100	PDTC, ATC Cohort selection depending driver mutation	II	Recruiting
Durvalumab, Tremelimumab, SBRT	ICI+ICI+SBRT	PD-L1 and CTLA-4	NCT03122496	ATC	I	Completed
Pembrolizumab, docetaxel/doxorubicin, radiation (2019) (115)	ICI+CT+RT	PD-1	NCT03211117	ATC	II	Completed

ICIs, immune checkpoints inhibitors; PD-1, programmed death protein-1; ATC, anaplastic thyroid cancer; CT, chemotherapy; RT, radiation therapy; TKI, tyrosine kinase inhibitors; RAIR, radioactive iodine refractory; DTC, differentiated thyroid cancer; SBRT, stereotactic body radiation therapy; VEGFR, vascular endothelial growth factor receptor; PDGFR, platelet-derived growth factor receptor; EGFR, epidermal growth factor receptor; FGFR, fibroblast growth factor receptor; PD-L1, programmed death-ligand 1; c-kit, mast/stem cell growth factor receptor kit; CTLA-4, cytotoxic T-lymphocyte associated protein.

Agents targeted on rarer drivers in ATC may also provide clinical efficacy though further studies are warranted, such as NTRK inhibition with larotrectinib or entrectinib (71, 73), mTOR inhibition with everolimus (90), ALK inhibition with Crizotinib (69, 70) or ceritinib and the RET inhibition with selpercatinib or pralsetinib (78, 79, 91).

### Peptide receptor radionuclide therapy

The term theranostics is the combination of diagnosis and therapy. The first and most classic application of this concept is radioactive iodine treatment performed on thyroid cancer patients since 1946. Recently, theranostics using radiolabeled somatostatin analogs have proved to be a milestone in the management of SSTR-expressing tumors. ^177^Lu-labeled or ^90^Y-labeled somatostatin analogs that bind somatostatin receptors are the most common PRRT in clinical practice. ^177^Lu-DOTATATE demonstrated modest efficacy of biochemical or anatomic response for RAIR-DTC patients (92, 93). In another study of ^90^Y-DOTATOC for RAIR-DTC patients (n=11), disease control was observed in 63.6% (7/11) patients (2 of PR and 5 of SD) with a duration of response of 3.5-11.5 months (94). Despite of heterogeneous response, PRRT may be a potential choice for RAIR-DTC with high expression of SSTRs owing to the efficacy and promising safety profile, and more large-scale studies are needed.

In recent years, FAP-targeted radionuclide therapy with ^177^Lu/^90^Y-labeled FAP inhibitors (FAPIs) have been reported as novel therapeutic options for refractory cancers, including pancreas, breast, and colorectal cancer. Most recently, a pilot study evaluated the efficacy of ^177^Lu-DOTAGA.(SA.FAPi)_2_ for RAIR-DTC patients who failed previous sorafenib/lenvatinib with ^68^Ga-DOTA.SA.FAPi uptake in PET/CT(n=15) (95). PR was documented in four (26.7%), and SD in three patients (20%); the serum Tg level significantly decreased after treatment. Another recent study reported a RAIR-DTC patient received SD after 4 circles of treatments of FAP-targeted radionuclide ^177^Lu-FAPI-46 (96). The results demonstrated that FAPi-based targeted theranostics might provide a novel treatment option for patients with advanced RAIR-DTC.

### Immunotherapy

The relationship between thyroid cancer and the immune system has long been studied owing to the common co-occurrence of papillary thyroid cancer and Hashimoto’s thyroiditis. The abnormality of the immune microenvironment and immune response partially contributes to DTC tumorigenesis and progression including the recruitment of immunosuppressive cells such as tumor-associated macrophages (TAMs), the expression of negative immune checkpoints, like programmed death-ligand 1 (PD-L1), cytotoxic T-lymphocyte associated protein (CTLA-4). PD-L1 was positively expressed in 6.1-53.2% of PTCs (97–99). The percentage increased to 61% in pT4 DTC and >70% in advanced-stage (III/IV) PTC, and 75%-80% in the ATC subset (97, 100). PD-L1 positive expression in PTC correlates with a greater risk of recurrence and shortened disease-free/overall survival (97, 101). Based on the above findings, immunotherapeutic strategy including immune checkpoint inhibitors (ICIs) may have a seat to manage advanced thyroid cancer. ICIs have two major classes: those targeting CTLA-4 such as ipilimumab and tremelimumab, and those targeting PD-1 such as nivolumab, pembrolizumab, spartalizumab or its ligand PD-L1 such as avelumab, atezolizumab, and durvalumab. ICIs act to enhance the effector T cells and inhibit the regulatory suppressor cells, and re-establish immune surveillance from which malignant cells are able to evade. Experience with ICIs in the treatment of RAIR-DTC is still limited.

A phase Ib KEYNOTE-028 trial of pembrolizumab enrolled 22 advanced thyroid cancer patients showed a manageable safety profile and clinical efficacy with PR of 9.1%, SD of 59.1%, and PD of 31.8% (102). The FDA approved the pembrolizumab for treatment of previously treated solid tumors with high tumor mutation burden in 2020 based on results of phase II KEYNOTE-158 trial, which included two patients with thyroid cancer (103).

It’s worth noting that the identification of immune biomarkers is important for patient selection. PD-L1 might have selective significance as a promising screening indicator for immune therapy (104, 105). A recent phase II single-arm study of spartalizumab, a PD-1 inhibitor, showed a favorable ORR of 29% in PD-L1 (+) vs. 0% in PD-L1 (–) ATC patients (n=42); the highest rate of response was observed in the subset of patients with PD-L1 ≥ 50% (6/17; 35%); median PFS and OS are 1.7 and 5.9 months, respectively; OS also correlated with PD-L1 status, with a median OS of 1.6 months in patients with PD-L1 < 1%, compared with not yet reached in PD-L1(+) patients (106). Notably, the co-existence of thyroid cancer with thyroiditis is common, and PD-L1 expression can also be detected in inflammatory thyroid tissue (107). Therefore, it should be cautious to interpret PD-L1 expression for thyroid cancer combined with thyroiditis and more investigations are needed.

#### Treatment combination including immunotherapy

Dual targeting of the immune system in the thyroid tumor microenvironment may, in theory, tone up the clinical benefits. Several clinical trials are ongoing to evaluate dual immunotherapy, such as PD-1 inhibitors (nivolumab) plus CTLA-4 inhibitor (ipilimumab), and PD-L1 inhibitor (durvalumab) plus CTLA-4 inhibitor (tremelimumab) **(** **Table 4****)**.

Immunotherapy combined with TKIs may also augment efficacy in ATC. In the preclinical model, both VEGF-A and BRAF^V600E^ are positively associated with upregulation of checkpoints expression, and the combination of BRAF^V600E^ inhibitor and anti-PD-L1 treatment reduced tumor burden significantly more than either single agent (108–110). Another preclinical study showed that anti-PD-1/PD-L1 therapy augments lenvatinib’s efficacy by favorably altering the immune microenvironment of murine ATC (111). Clinically, in a case report, an ATC patient with BRAF and PD-L1 positivity was treated with vemurafenib and nivolumab, the patient continues to be in complete remission for 20 months after initiation of treatment (112). A combination of lenvatinib and pembrolizumab also showed promising efficacy for ATC (n=6) with CR of 66.6%, SD of 16.7%, and PD of 16.6%; the median OS was 18.5 months with three ATC patients being still alive without relapse (40, 27, and 19 months) (113). More phase II studies are currently assessing the effect of combining MKIs with immune therapy, such as pembrolizumab plus lenvatinib, nivolumab plus encorafenib/binimetinib **(** **Table 4****)**.

A preliminary study evaluated RAI and anti-PD-L1 agent durvalumab in recurrent/metastatic thyroid cancer based on the hypothesis that RAI can enhance the presentation of thyroid protein immunogens and the putative neoantigens may amplify the effectiveness of ICIs. In a preliminary trial, eleven recurrent/metastatic thyroid cancer patients were treated with durvalumab and RAI (100 mCi); two patients had PR, 7 had SD, and 2 had PD (114).

Albeit disappointing outcome in a phase II study of pembrolizumab combined with chemoradiotherapy as initial treatment for anaplastic thyroid cancer, other combination strategies, such as ICIs plus SBRT and ICIs plus chemotherapy are ongoing (115) **(** **Table 4****)**.

## Challenges and perspectives

Over the past few years, the understanding of the underlying molecular mechanisms involving thyroid dedifferentiation and the identification of key disease-causing driver genes have led to the introduction of several new radionuclide imaging. Some mutations such as EIF1AX, IDH1/IDH2, and other signaling pathways concerning the dedifferentiation process are still not clear, the importance of which needs to be clarified. Several studies have found that glycosylation, acetylation, methylation, and ubiquitination are closely related to the epigenetics of oncogenesis (116). There are also some studies involving these proteomic analyses in thyroid tumorigenesis (6, 16, 26). But the molecular mechanisms remain unknown, and more studies are needed. Additionally, clinical evaluation of functional imaging of dedifferentiated thyroid cancer has shown the potential of disease diagnosis and treatment, and more studies related to molecular targeted probes are required for the diagnostic and even therapeutical purposes of dedifferentiated thyroid cancer in the future.

The emergence of new targeted therapy has undoubtedly provided us with more treatment options for advanced thyroid cancer. The upcoming results of the phase III trials of anlotinib and donafenib are expected to provide new options for the management of advanced thyroid cancer. But how to properly use these “news weapons” is worthy of attention, whether as a supplement to or complete subversion of the current standard treatment mode. The timing of the initiation with novel agents is of vital significance. Should the intervention be administrated at an early stage or be waited as the last resort of salvage treatment? Moreover, considering the relatively slow rate of disease progression for most RAIR-DTC, how to balance the benefit of PFS/OS and the quality of life of patients? More explorations and investigations are needed to address these issues. In the future, well-designed clinical trials especially head-to-head studies will help understand the comparative efficacy of novel agents. And it’s of pivotal significance to identify an appropriate sequential and combined treatment strategy to minimize cross-resistance or exposure to inactive drugs in the long clinical course for advanced thyroid cancer.

## Conclusion

The overall prognosis of thyroid cancer is favorable yet the treatment of advanced thyroid cancer patients remains challenging. Thyroid cancer is a heterogeneous disease driven by variable molecular alterations. Over the past decade, advances in the understanding of oncogenic alterations and signaling pathways have helped clinicians diagnose and early recognize potential advanced thyroid cancer patients. The findings of molecular modifications involving DNA methylation, protein post-translational modification such as phosphorylation, acetylation, ubiquitination, and glycosylation also alter the therapeutic strategy for advanced thyroid cancer. Furthermore, the growing number of molecular imaging studies provide more potential for the diagnosis and treatment of advanced thyroid cancer. Targeted therapy, immunotherapy, and theranostic are making robust progress in the personalized management of advanced thyroid cancer. Further investigations and more real-world clinical outcomes are warranted to develop more effective targeted therapies, and select candidate patients who might benefit and improve the treatment modalities of advanced thyroid cancer.

## Author contributions

All authors contributed to the conception and design of the study and to data acquisition and analysis. The first draft of the manuscript was written by JL and YZ. FS contributed to the investigation and resources. LX and XS reviewed and edited the manuscript. All authors contributed to the article and approved the submitted version.

## Funding

This work was financially supported by grants from the Shandong Provincial Natural Science Foundation (ZR2021LZL005), the Start-up fund of Shandong Cancer Hospital (2020PYA04), and the Shandong Provincial Natural Science Foundation (ZR2019PH051).

## Conflict of interest

The authors declare that the research was conducted in the absence of any commercial or financial relationships that could be construed as a potential conflict of interest.

The handling editor declared a shared affiliation, though no other collaboration, with the authors at the time of the review.

## Publisher’s note

All claims expressed in this article are solely those of the authors and do not necessarily represent those of their affiliated organizations, or those of the publisher, the editors and the reviewers. Any product that may be evaluated in this article, or claim that may be made by its manufacturer, is not guaranteed or endorsed by the publisher.
